# The modulation of iron metabolism affects the Rhabdomyosarcoma tumor growth in vitro and in vivo

**DOI:** 10.1007/s10238-023-01012-5

**Published:** 2023-02-10

**Authors:** Michela Asperti, Luca Cantamessa, Magdalena Gryzik, Mattia Bugatti, Silvia Codenotti, Andrea Denardo, William Vermi, Alessandro Fanzani, Maura Poli

**Affiliations:** 1 Department of Molecular and Translational Medicine, University of Brescia, 25123 Brescia, Italy; 2 Unit of Pathology, ASST Spedali Civili Di Brescia, 25100 Brescia, Italy

**Keywords:** Iron, Rhabdomyosarcoma, Iron supplementation, Iron chelators

## Abstract

Rhabdomyosarcoma (RMS) is an aggressive rare neoplasm that derives from mesenchymal cells, which frequently develops resistance to the current therapies and the formation of metastases. Thus, new therapies are needed. The alteration of iron metabolism in cancer cells was effective in reducing the progression of many tumors but not yet investigated in RMS. Here we investigated the effect of iron modulation in RMS both in vitro and in vivo. We first characterized the most used RMS cell lines representing the most common subtypes, embryonal (ERMS, RD cells) and alveolar (ARMS, RH30 cells), for their iron metabolism, in basal condition and in response to its modulation. Then we investigated the effects of both iron overload and chelation strategies in vitro and in vivo. RMS cell lines expressed iron-related proteins, even if at lower levels compared to hepatic cell lines and they are correctly modulated in response to iron increase and deprivation. Interestingly, the treatment with different doses of ferric ammonium citrate (FAC, as iron source) and with deferiprone (DFP, as iron chelator), significantly affected the cell viability of RD and RH30. Moreover, iron supplementation (in the form of iron dextran) or iron chelation (in the form of DFP) were also effective in vivo in inhibiting the tumor mass growth both derived from RD and RH30 with iron chelation treatment the most effective one. All the data suggest that the iron modulation could be a promising approach to overcome the RMS tumor growth. The mechanism of action seems to involve the apoptotic cell death for both iron supplementation and chelation with the concomitant induction of ferroptosis in the case of iron supplementation.

**Supplementary Information:**

The online version contains supplementary material available at 10.1007/s10238-023-01012-5.

## Introduction

Rhabdomyosarcoma (RMS) is an aggressive neoplasm that derives from mesenchymal cells which failed to undergo complete myogenic differentiation. Globally, it has a frequency of about 4, 5 patients per million individuals, and it mostly occurs in children from 0 to 4 years old and adolescents aged between 15 and 19 years. However, due to the rarity of the disease, its epidemiology remains uncertain [1]. According to the histological characteristics from patients’ samples, four RMS subtypes have been distinguished: embryonal (ERMS, which develops most frequently in the neck and head region and in the genitourinary tract), alveolar (ARMS, which mostly develops in extremity sites), pleomorphic (a very rare subtype occurring typically in adults) and spindle-cell RMS (a rare variant that occurs in children). The most common subtypes, ERMS and ARMS, occur in the pediatric age, and account for 60% and 20% of RMS cases, respectively [2]. The mutational landscape of the embryonal subtype is characterized by various point mutations and karyotypic alterations such as point mutations in the *TP53* or the *FGFR4* genes, loss of heterozygosity on various loci or whole chromosome losses or gains, while ARMS frequently presents chromosomal translocations that lead to the fusion of the *PAX3* or *PAX7* gene with the *FOXO1* gene, creating a chimeric oncogene [3]. In both cases, these modifications lead to the activation of various signaling pathways involved in cell proliferation and associated with the tumor aggressiveness, including the extracellular signal regulated kinase 1/2 (ERK 1/2), the WNT and the mTOR pathways [4, 5]. Since not all ARMS tumors present the fusion gene, with the aggressiveness resembling more the embryonal subtype, RMS has been reclassified in fusion-positive (FPRMS) with the *PAX3/7-FOXO1* chimeric gene and fusion-negative RMS (FNRMS), harboring the mutational burden typical of ERMS. This classification better fits with prognostic values, with FP tumors being the most aggressive and less curable ones [6–8].

The first line treatment for RMS is surgical resection, which is often followed by chemotherapy and/or with co-adjuvant radiotherapy, used mostly when complete resection is not possible [9]. Unfortunately, RMS frequently develops resistance to the current therapies, which, together with the formation of metastases, often already present in undetectable sizes in newly diagnosed cases, constitute the main reason of therapy failure [10, 11]. Thus, of importance is to find new targets, and many different strategies have been currently tested both in in vitro and in in vivo models. However, due to the rarity of this neoplasm, only few clinical trials have been approved, therefore making difficult to establish the efficacy of those treatments [12]. Among the various antitumoral approaches, the alteration of iron metabolism in cancer cells has demonstrated to be effective in reducing the progression of many tumor types [13].

Iron is a micronutrient that is essential for all life forms and is involved in a number of biological processes like DNA synthesis and repair, energy production in mitochondria and the generation of heme groups and Fe-S clusters [14]. Iron is imported into the cell by transferrin receptor 1 (TfR1)-mediated endocytosis, it is reduced to its ferrous (Fe^2+^) form by the protein STEAP3 and then it is transported to the cytoplasm by divalent metal transporter 1 (DMT1) [15]. The cytosolic free ferrous ions constitute the Labile Iron Pool (LIP), which is the source of iron bioavailable for the incorporation in proteins and as a cofactor for several enzymes. High LIP levels can be also toxic for cells since iron interacts with hydrogen peroxide through the Fenton reaction, leading to the production of free radicals and thus to the oxidative stress. The iron excess is stored in ferritin (Ft), a 24-meric protein responsible for the storage of iron in the non-toxic form of ferric (Fe^3+^) ions [16]. Iron can also be exported through the transmembrane channel ferroportin (FPN), the only known iron exporter [17]. In cancer cells, higher levels of iron sustain their accelerated metabolism. Increased TfR1 level allows greater iron uptake, and, at the same time, decreased FPN and ferritin levels limit iron efflux from the cell and the amount of biologically available iron, respectively. All these metabolic changes lead to the specific iron-dependence named “iron addiction” of cancer cells, a peculiarity which is studied in various tumor types and that could be used as target for an anti-cancer strategy [18]. There are two alternative ways to take advantage of iron dysregulation in cancer. The first one exploits iron chelators in order to reduce the amount of iron available for cellular metabolism. Alternatively, a treatment with iron can promote the Fenton reaction and the production of ROS, an approach named “iron overload” induction [19]. Many iron modulators have been proposed in the context of anti-cancer therapy, such as the iron chelators Deferiprone (DFP), Deferrioxamine (DFO), Deferasirox which were effective in preclinical studies of pancreatic, breast, liver, gastric, hepatic and esophageal cancers [15]. On the other hand, the use of iron supplementation as anti-cancer strategy is still controversial since iron could promote tumorigenicity and resistance to the therapies [15]. However, some works showed that the use of iron oxide nanoparticles is effective against mammary tumor [20], and that the combination of iron and some chemotherapeutic agents is effective in potentiating the anti-tumor effect in prostatic cancer [21] and multiple myeloma [22, 23].

To date, iron metabolism has been poorly studied in RMS, since this neoplasm is not derived from tissues directly involved in systemic iron handling.

Therefore, in this work we characterized some of the most used RMS cell lines representing ERMS and ARMS subtypes [24, 25] for their iron metabolism, and investigated the effects of both iron overload and chelation strategies both in vitro and in vivo. It has been observed that the analyzed cell lines expressed detectable levels of iron-related genes or proteins. Moreover, the iron manipulation was effective in counteracting RMS tumors, reducing the tumor growth of both ERMS and ARMS.

## Materials and methods

### Antibodies and chemicals

Antibodies used: anti-TfR1 (Cod. 136,800, Thermo Scientific, Waltham, MA), anti-Ferroportin (Cod. NBP1-21,502, Abnova, Novus Biologicals, Littleton, CO), and anti-Tubulin (Cod. T5168, Sigma-Aldrich, Saint Louis, MO). HRP-conjugated secondary antibodies used: anti-mouse (Cod: sc-516102, Santa Cruz Biotechnology, Dallas, TX) and anti-rabbit (Cod. A120-101P, Bethyl Laboratories, Inc., Montgomery, TX).

The chemicals dissolved in sterile deionized water: ferric ammonium citrate (FAC, Cod. F5879, Sigma-Aldrich, Saint Louis, MO), ascorbic acid (no. A4034, Sigma-Aldrich, Saint Louis, MO), Bathophenanthroline disulfonic acid disodium salt hydrate (BPS, Cod. B1375, Sigma-Aldrich, Saint Louis, MO), Desferoxamine (DFO, Cod. S0080A, Novartis), Deferiprone (DFP, kind gift of Prof. P. Ponka, University of Montreal, Canada); 3-[4, 5-dimethyl-2- thiazolyl]-2, 5-diphenyl-2H-tetrazolium bromide (MTT, Cod. M5655, Sigma-Aldrich, Saint Louis, MO).

Iron dextran (Cod. D851, Sigma-Aldrich, Saint Louis, MO) was diluted in saline buffer.

### Cell culture

The human Embryonal Rhabdomyosarcoma cell lines (ERMS: RD, SMS and RH36) and the human Alveolar Rhabdomyosarcoma cell lines (ARMS: RH4, RH18 and RH30) were cultured in Dulbecco’s Modified Eagle Medium (DMEM, Gibco, Life Technologies, Carlsbad, CA) supplemented with 10% endotoxin-free fetal bovine serum (Gibco, Life Technologies, Carlsbad, CA), 0.04 mg/mL gentamicin (Euroclone, Milan), 2 mM L-glutamine (Euroclone, Milan) and 1 mM sodium pyruvate (Euroclone, Milan) and maintained at 37 °C in 5% CO_2_.

### Cell viability assay

Cell viability was evaluated with MTT assay. In brief, cells were plated in a 96-well plate (at a density of 1.5 × 10^3^ cells for RH30; 2.0 × 10^3^ cells for RH4; 2.5 × 10^3^ cells for RD; 3.0 × 10^3^ cells for SMS and RH18; 4.0 × 10^3^ cells for RH36) and exposed to various concentrations of the different chemicals. After 24/48/72 h treatment, the supernatant was removed and 100 µl of MTT solution (0.5 mg/mL) diluted in cell medium were added. After 3.5 h of incubation at 37 °C and 5% CO_2_ the insoluble formazan was dissolved with 75 µL of DMSO. Plates were shaken for 15 min at 37 °C until complete dissolution and absorbance was measured at 540 nm wavelength using a Multiskan©EX plate reader (Thermo Scientific, Waltham, MA).

### Western blot analysis

Western blot was used to analyze protein expression. In brief, after extraction, protein concentration was quantified using Micro BCA™ Protein Assay Kit (Pierce). Equal amounts of protein homogenates were boiled at 99 °C for 5 min before separation by SDS–polyacrylamide gel electrophoresis and transferred to a polyvinylidene fluoride (PVDF) membrane (GE, Carlsbad, CA). Membranes were blocked for 30 min at 37 °C with Tris-buffered saline with 0.1% Tween-20 (TBS-T) with 2% milk and incubated overnight at 4 °C with the primary antibodies. Following TBS-T wash, membranes were incubated with HRP-conjugated secondary antibodies for 1 h at RT. Membranes were washed again in TBS-T prior to signal visualization using enhanced chemiluminescence (PDS kit, Protein Detection System, GeneSpin, Milan, IT). The obtained bands were quantified using densitometry analysis (ImageJ Software).

### RNA extraction and quantitative qRT-PCR

Total cell RNA was recovered with TRI Reagent (Sigma-Aldrich, Saint Louis, MO), according to the manufacturer’s instruction. cDNA was generated by reverse transcription using 1.5 µg RNA and ImProm-II™ Reverse Transcription System kit (Promega, Madison, WI) according to the manufacturer’s instructions. Samples (1.5 µL) were used for quantitative reverse transcription polymerase chain reaction (qRT-PCR) assay, using PowerUp SYBR Green Master Mix (Life Technologies), according to the manufacturer’s instructions.

Primers used were:

Hs HAMP1 Forward 5'-CCAGCTGGATGCCCATGTT-3' and Reverse 5'-GCCGCAGCAGAAAATGCA-3'; Hs NCOA4 Forward 5′-CTTTGGGCCGTAGGTTAGTG-3′ and Reverse 5′-GTTCTCTATTACTGGAGCTGCC-3′; Hs ZIP14 Forward 5'- CCTGCTTGGCTTATGGAGAA -3' and Reverse 5'- CCTCGCCATACCGATGTATTAG -3'; Hs GAPDH Forward 5'- GGTGTGAACCATGAGAAGTATGA -3' and Reverse 5'- GAGTCCTTCCACGATACCAAAG -3'.

All data were normalized to GAPDH expression and expressed as 2^−ΔCt^.

### ELISA assay

The 96 wells plates were coated with 10 μg/mL of primary antibody against L-ferritin (LFO3) or H- ferritin (RH02) (diluted in 50 mM carbonate buffer pH 9.6) for overnight at 4 °C. After three washes with PBST (phosphate buffer saline with 0.1% Tween20), the wells were over-coated by adding 3% bovine serum albumin (BSA) diluted in PBS for 1 h at 37 °C. After washing with PBST, 50 μg of protein extract diluted in 1% BSA-PBST for both L- and H-ferritin analysis were aliquoted in duplicate and incubated at 37 °C for 2 h. A standard curve using recombinant human L- or H-ferritin was added, as calibrator. After three washings in PBST, 0.1 mL of anti-L or H-ferritin antibody HRP labelled (diluted 1:500 and 1:200 in 1% BSA-PBST, respectively) were added and plate incubated for 1 h at 37 °C. After three washings in PBST, HRP activity was detected using 1 mg/mL tetramethylbenzene (TMB) dissolved in dimethyl sulfoxide (DMSO) and diluted 1:10 in phosphate-citrate buffer pH 5 with added fresh hydrogen peroxide to final concentration 0.006% and the absorbance read at 620 nm by Multiskan©EX plate reader (Thermo Scientific, Waltham, MA). The reaction was stopped by adding 1 N sulphuric acid and the absorbance was measured at 405 nm using a Multiskan©EX plate reader (Thermo Scientific, Waltham, MA). The concentration of ferritins was extrapolated from the calibrator curve and expressed as ng of ferritin/mg of protein extract.

### Calcein-AM assay

RD or RH30 cells (1.2 × 10^4^) were seeded on 96-well plates and treated with 100–500 μM FAC (for RD) or 50–100 μM FAC (for RH30) or 100–200 μM DFP for 16 h. The cells were incubated with 0.25 μM Calcein-AM in DMEM with 1 mg/mL BSA for 1 h at 37 °C. After washing, 100 μL of 1X HBSS was added, and the fluorescence monitored at an excitation of 488 nm and an emission of 517 nm using EnSight Multimode plate reader (PerkinElmer, Waltham, MA). Cells were then fixed in 4% PFA, stained with Crystal violet solution (0.1% Crystal violet, 20% methanol) and the absorbance at 540 nm used as normalization. The data were expressed as ratio Calcein-AM Fluorescence/Crystal violet absorbance. The Calcein-AM quenching is inversely proportional to the iron concentration.

### Wound healing assay

2.5 × 10^5^ RD or RH30 cells were seeded in 6-well plates and when they reached 90–100% of confluence, a scratch was made through the cell monolayer using a sterile micropipette tip. After washing twice with sterile PBS to remove cell debris, the culture medium was added with or without FAC (500 µM for RD and 100 µM for RH30) or 100 µM DFP (for both RD and RH30). After 16 h for RD and 10 h for RH30, cells were washed gently with cold PBS and fixed for 15 min with 2.5% glutaraldehyde solution. After gently washing with PBS, the cells were stained with Crystal Violet solution (0.1% Crystal violet, 20% methanol) for 15 min under stirring. The dye was then removed, and the plates washed in running water. The images of the wounds were acquired under the microscope (Leitz LABOVERT microscope with Sony HDMI camera Mod. Md6is). The migration abilities were quantified by measuring the area of the scratched regions using the ImageJ software.

### Clonogenic forming assay

RD and RH30 cells were seeded at 1 × 10^3^ and 5 × 10^2^ cells/well, respectively, in dishes Ø 3.5 cm. After 48 h, cells were treated with different concentration of FAC or DFP. After 48 h, medium was replaced with fresh one and cells cultured for 5 days, until well-defined colonies had formed. Cells were washed in PBS, fixed in 3% paraformaldehyde (PFA) for 15 min at RT and stained with Crystal Violet solution (0.2% Crystal violet, 20% methanol) for 10 min at RT. The dishes were washed with deionized water and representative pictures of the colonies were taken. The dye was solubilized in 1% sodium dodecyl sulfate (SDS)/PBS solution and the absorbance measured at 540 nm using a Multiskan©EX plate reader (Thermo Scientific, Waltham, MA).

### ROS measurement

The reactive oxygen species (ROS) production was measured using CM-H2DCFDA (Molecular Probe), according to the manufacturer’s instructions. Briefly, RD or RH30 cell lines (2 × 10^4^ cells/well) were plated in 96-well black plate and after 24 h treated with 500 μM FAC or 100–200 μM DFP for 24–48–72 h. After treatments, media was changed with media containing 1 μM ROS probe (100 μL/well) and incubated for 30 min at 37 °C protected from light. Media was then removed and replaced with fresh media and the fluorescence (RFU) was monitored by the EnSight Multimode plate reader (PerkinElmer, Waltham, MA), at Ex/Em = 495/529 nm. The Cells were then fixed in 4% PFA, stained with Crystal violet solution (0.1% Crystal violet, 20% methanol) and the absorbance at 540 nm used as normalization. The data were expressed as ratio ROS fluorescence/Crystal violet absorbance.

### Lipid peroxidation assay

The RD or RH30 cells were seeded on glass coverslips (5 × 10^5^) and treated with 100–500 μM FAC (for RD) or 50–100 μM FAC (for RH30) or 100–200 μM DFP for 16 h. The cells were incubated with 2.5 μM C11-BODIPY^581/ 591^ for 30 min at 37 °C, fixed with 4% PFA for 15 min at RT and stained with 0.1 μg/mL solution of DAPI dye. The images were acquired using Zeiss Axiovert microscope and SensiCam-PCO Optics (GmbH, Germany).

### MitoSOX™ red mitochondrial superoxide indicator assay

RD cells were seeded at 2.5 × 10^5^ in 6-well plates and 24 h after seeding, treated with 500 µM FAC or 100–200 µM DFP. After 24–48–72 h, cells were collected and labelled with 5 µM MitoSOX™ (Molecular Probes) diluted in medium and incubated in the dark for 20 min at 37 °C. Cells were then washed and suspended in medium and fluorescence detected by cytofluorimeter instrument (MACSQuant Analyzer, Miltenyi Biotec).

### Annexin V

RD cells were seeded at 2.5 × 10^5^ in 6-well plates and 24 h after seeding, treated with 500 µM FAC or 100 µM DFP. After 24–48–72 h, cells were washed twice with 1X PBS, resuspended in 1X Annexin V Binding Buffer, from the kit Apoptosis Detection Kit FITC, Immunostep) at the concentration of 10^6^ cells/mL, labelled with 5 µL Annexin V-FITC and 5 µL Propidium Iodide (PI) and incubated for 15 min at RT in the dark. Then 400 µL Annexin V Binding Buffer 1X were added and cells were analyzed by cytofluorimeter instrument (MACSQuant Analyzer, Miltenyi Biotec).

### Measurement of caspase-3, caspase-8 and caspase-9

The Caspase-3, -8 and -9 activities were assessed using the fluorometric kit Caspase 3 Multiplex Activity Assay (Abcam), according to the manufacturer’s instructions.

Briefly, the RD or RH30 cells (2 × 10^4^ cells/well) were plated in 96-well black plate and after 24 h treated with 500–1000 µM FAC or 100–200 µM DFP for 3–6–24–48–72 h. To assay the Caspases activity in each well, an Assay Loading Solution was prepared by adding 50 µL of substrate (Caspase-3, -8, -9) to 10 mL of Assay buffer. Then, 100 µL of the Caspases Assay Solution were added to each well, without removing culture media. The plate was incubated at room temperature for 60 min, protected from light and the fluorescence (RFU) was monitored by the EnSight Multimode plate reader (PerkinElmer, Waltham, MA), at specific wavelengths, as follow: Ex/Em = 535/620 nm (Caspase-3), Ex/Em = 490/525 nm (Caspase-8), Ex/Em = 370/450 nm (Caspase-9).

### In vivo tumor xenograft

To generate murine subcutaneous tumor, 7 weeks-old male NOD/SCID mice were subcutaneously inoculated in the flank with 5 × 10^6^ RD or RH30 cells (representative ERMS or ARMS, respectively), resuspended in 100 μL of saline buffer. 5 days after injection, mice were randomly divided in three groups and treated as follows: Vehicle (100 μL saline buffer intraperitoneally ip), iron dextran (200 mg/kg of iron ip, that correspond to 5 mg iron/mouse, at days 5, 20, 40 after tumor challenge for RD and 100 mg/kg of iron ip, that correspond to 2.5 mg iron/mouse, at days 5, 14, 21, 28 after tumor challenge for RH30) or with Deferiprone (DFP, 1 mg/mL dissolved in drinking water with access ad libitum). In detail, for RD 6 mice untreated, 4 mice treated with iron dextran, 8 mice treated with DFP; for RH30 8 mice untreated, 10 mice treated with iron dextran, 6 mice treated with DFP).

The tumor dimension was measured by a microcaliper at 15, 27, 36, 43 and 49 days after RD tumor challenge and at 19, 25, 31, 41 after RH30 tumor challenge and the volume was calculated using to the formula V = (D × d^2^)/2, where D and d are the major and minor perpendicular tumor diameters, respectively and expressed as Tumor volume (mm^3^). At day 49 for RD and day 41 for RH30, mice were sacrificed and tumor, blood, liver and spleen harvested for further analysis.

Serum iron was determined spectrophotometrically with a commercial kit, according to the manufacturer’s instruction (Cod. SI257, Randox Laboratories), whereas non-heme iron content in spleen and liver was measured spectrophotometrically as previously described [26]. The study was approved by the Institutional Animal Care and Use Committee of the University of Brescia, Italy.

### Immunohistochemistry and digital analysis

Four-micron thick tissue sections were used for immunohistochemical staining. After antigen retrieval sections were incubated with anti-PECAM-1 (goat, clone M-20, rabbit polyclonal, diluted 1:200 from Santa Cruz Biotechnology) and anti-KI67 (rabbit, clone 30–9, ready to use from Ventana). Reactions were revealed using Goat-on-Rodent-HRP-Polymer (BIOCARE) or EnVision + System HRP Labelled polymer anti-Rabbit (Dako, Agilent) followed by DAB. Slides were digitalized using ScanScope CS Slide Scanner (Aperio Technologies, Leica) at 20 × magnification and analyzed by ImageScope. PECAM and KI67 analysis were obtained using Positive Pixel count V9 and Nuclear algorithm, respectively.

### Lipid peroxidation assay (MDA)

The relative concentration of MDA in the tumor xenografts lysates was determined using the Lipid Peroxidation (MDA) Assay kit (Abcam), following the manufacturer’s instructions. Briefly, about 20 mg of tumor tissue was homogenized in 303 µL of MDA Lysis Buffer, provided by the kit. The insoluble fraction was removed after centrifugation and the supernatant analyzed. The sample supernatants were mixed with the thiobarbituric acid (TBA) reagent and incubated at 95 °C for 1 h. The MDA in the samples reacts with the TBA forming MDA-TBA adduct, and the fluorescence (RFU) was quantified in 96-well plates using the EnSight Multimode plate reader (PerkinElmer, Waltham, MA) at Ex/Em = 532/553 nm. The data are expressed as nmol MDA normalized for mg of tumor tissue (nmol/mg tumor tissue).

### Statistical analysis

Data are presented as mean ± standard error of mean (SD). Statistical significance was assessed by one or Two-way ANOVA or unpaired Student’s *t* test, as reported in the captions, performed by GraphPad Prism 7 (GraphPad Software, Inc., La Jolla, CA). *P* values < 0.05 were considered as significant and reported in the graphs and captions.

## Results

### Rhabdomyosarcoma cell lines expressed iron-related proteins and genes and responded to iron supplementation and deprivation.

The “iron addiction” of tumor cells is a well described phenomenon for many types of tumors. The main features are: altered iron absorption, efflux and storage mechanisms, compared to the healthy counterpart, leading to an overall increase in intracellular iron, on which cancer cells depend for their growth. Despite this, the iron metabolism in some tumors, such as sarcomas has been little investigated. Thus, the present work characterized the main proteins and mRNAs associated to iron metabolism in rhabdomyosarcoma (RMS), which is a pediatric myogenic malignancy consisting of two main histotypes: embryonic (ERMS) and alveolar (ARMS).

To this aim, three cell lines representing ERMS (RD, SMS, RH36) and three cell lines representing ARMS (RH4, RH18, RH30) have been evaluated for the expression of Transferrin Receptor1 (TfR1, involved in iron uptake), Ferroportin (FPN, the sole cellular iron exporter), H- and L- ferritin proteins (responsible for iron storage) and of Zip14 (a zinc transporter which transports also iron), NCOA4 (the cargo receptor for ferritin degradation, in a process called ferritinophagy) and Hepcidin (the small hormone peptide involved in systemic iron regulation which binding FPN induce its degradation) mRNAs.

**Fig. 1:**
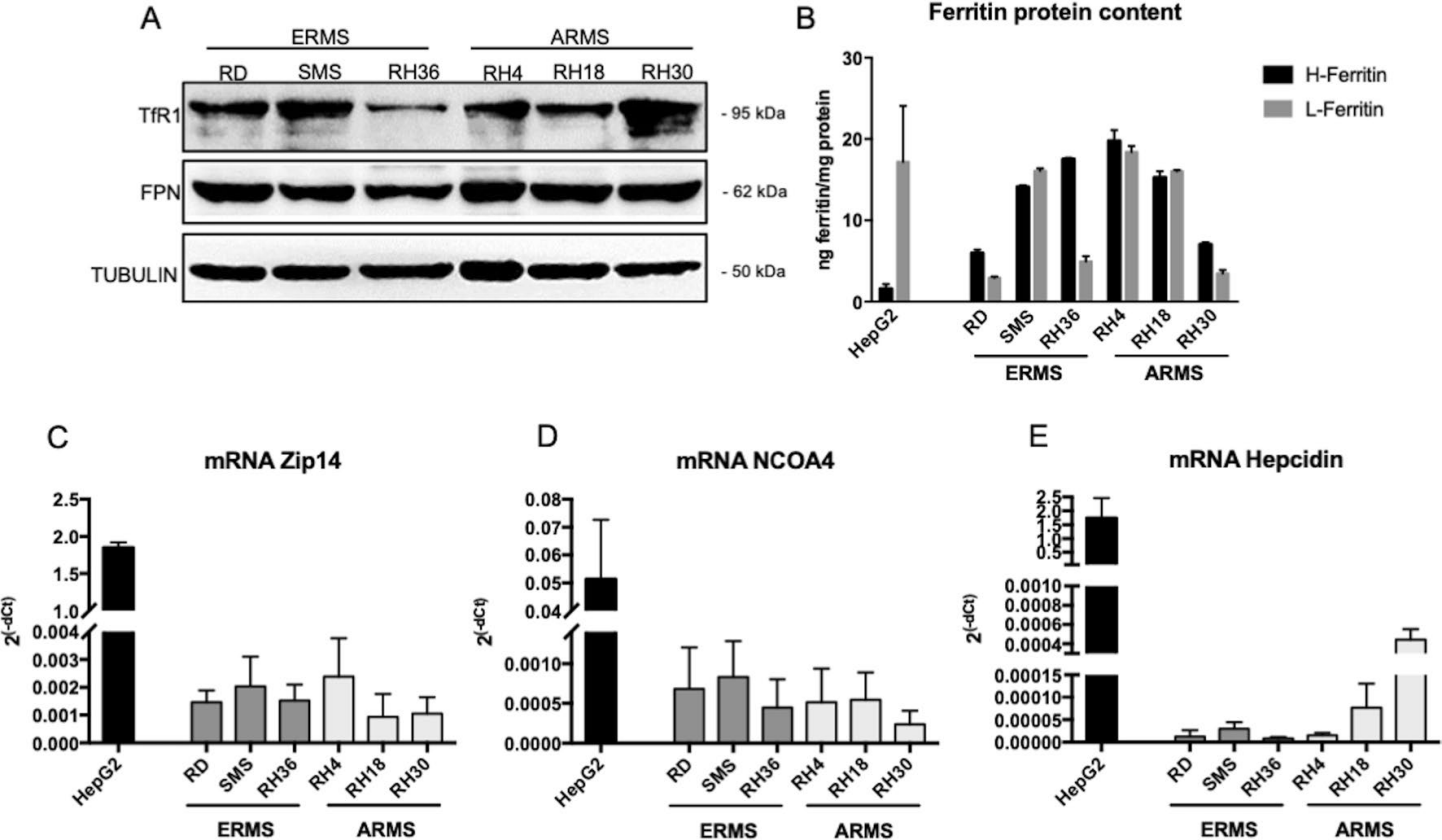
Rhabdomyosarcoma cell lines expressed iron-related proteins and genes. **A** Western blot analysis for Transferrin Receptor1 (TfR1), Ferroportin (FPN) in cell lines representing human Embryonal (ERMS: RD, SMS, RH36) and Alveolar (ARMS: RH4, RH18, RH30) rhabdomyosarcoma. Tubulin was used as loading control (*N* = 3). **B** ELISA assay for H- and L- ferritin in cell lines representing human ERMS and ARMS (*N* = 3). **C–E** qPCR for Zip14 (**C)**, NCOA4 (**D**) and Hepcidin (**E**) mRNAs in cell lines representing human ERMS and ARMS. The mRNA levels are expressed as 2^(−dCt)^ related to GAPDH (*N* = 3). HepG2 cell line was used as internal control for the analysis in B, C, D, E, since it is derived from liver, one of the main tissues involved in iron metabolism

As reported in Fig. 1A both ERMS and ARMS cell lines expressed detectable levels of TfR1 and FPN. In addition, it has been observed that both ERMS and ARMS cell lines showed detectable levels of both H- (black histograms) and L- (grey histograms) ferritin, with some heterogeneity among the histotypes (Fig. 1B). As reference, the content of both ferritins was evaluated in hepatocellular carcinoma HepG2 cells, a tumor derived from liver, one of the main organs involved in iron metabolism and well characterized for it. HepG2 cells expressed higher level of L-ferritin than H-ferritin (~ 17 ng L-ferritin/mg protein vs. ~ 2 ng H-ferritin/mg protein), as observed also in liver, since this subunit is mainly involved in iron nucleation in ferritin shell, whereas RMS cell lines expressed high levels also of H-subunit, which due to its ferroxidase activity, is involved in preventing the oxidative damage.

Finally, ERMS and ARMS cell lines expressed also ZIP14 (Fig. 1C), NCOA4 (Fig. 1D) and Hepcidin (Fig. 1E) mRNA, even if at lower levels compared to HepG2 cell lines.

Thus, RMS cells lines expressed iron-related proteins and genes for the maintenance of proper intracellular iron, necessary to sustain tumor cell proliferation.

**Fig. 2:**
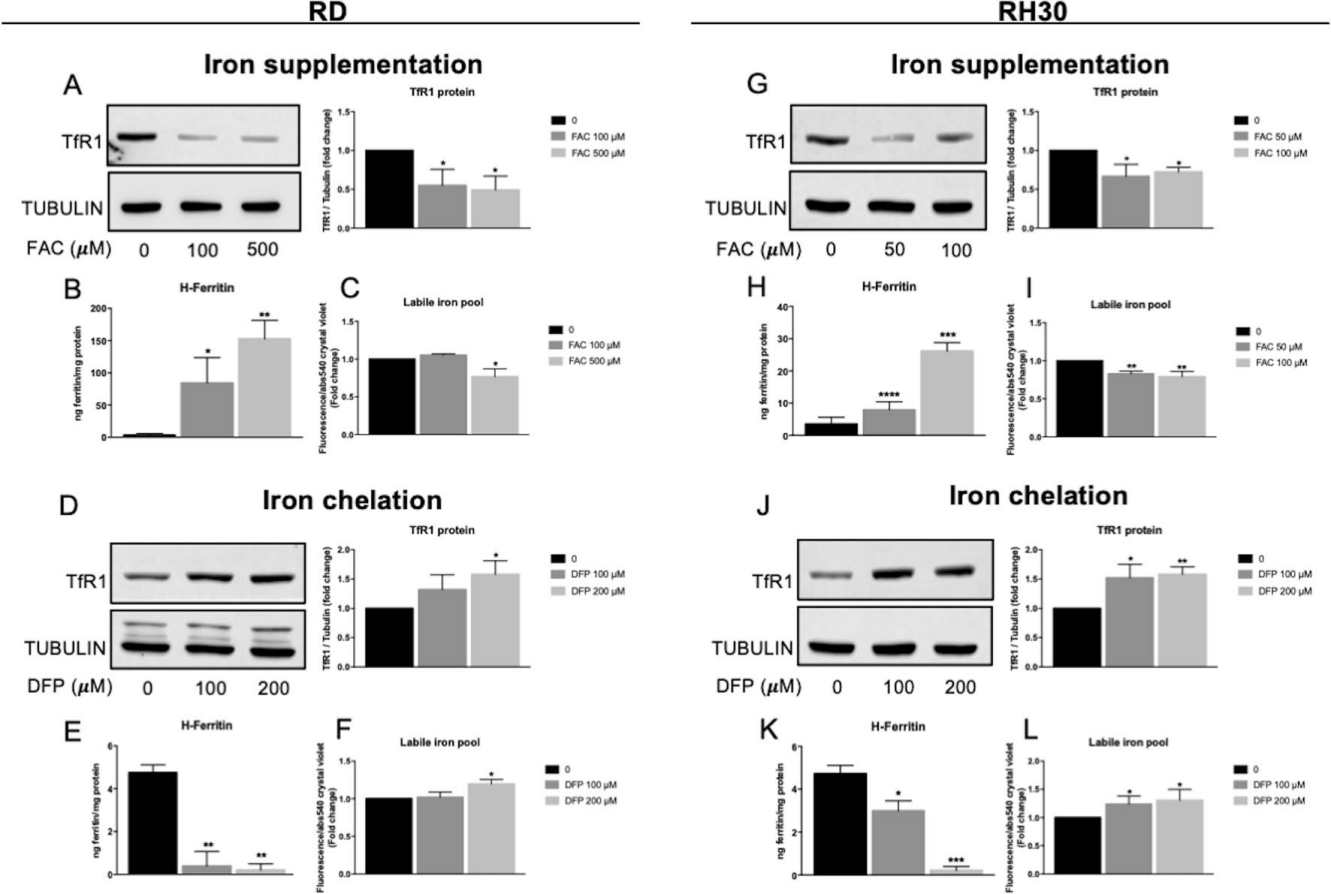
Short-term iron supplementation or iron chelation affected TfR1, ferritin and Labile Iron Pool. RD and RH30 cell lines treated with 100–500 µM or 50–100 µM of ferric ammonium citrate (FAC), respectively, or 100–200 µM Deferiprone (DFP) for 16 h. A, D, G, J) Western blot analysis for Transferrin Receptor1 (TfR1) in RD and RH30 cell lines. Tubulin was used as loading control. Densitometric analysis were performed using ImageJ software and data expressed as fold change over the untreated control cells (0) (*N* = 3). B, E, H, K) ELISA assay for H-ferritin in RD and RH30 cell lines (*N* = 3). C, F, I, L) Calcein-AM assay for the detection of Labile iron pool (LIP) in RD and RH30 cell lines. The values are expressed as ratio between the fluorescence detected and the absorbance at 540 nm after Crystal violet normalization. The differences were considered as significant for: **P < 0.01, *P < 0.05

To verify if the iron manipulation has an impact on iron-related proteins and on the intracellular iron content, the RD (among the Embryonal Rhabdomyosarcoma cell lines previously used) and RH30 (among the Alveolar Rhabdomyosarcoma cell lines previously used) have been treated with Ferric Ammonium Citrate (FAC, 100–500 µM for RD and 50–100 µM for RH30) or Deferiprone (DFP, 100–200 µM) for 16 h and iron content (in the form of labile iron pool, LIP), TfR1 and H-ferritin protein content were analyzed, as the main proteins involved in iron up-take and storage, respectively. As shown in Fig. 2, in response to iron supplementation TfR1 decreased (Fig. 2 A and G), the H-ferritin content increased (Fig. 2 B and H) as well as the LIP (Fig. 2 C and I) in both cell lines. Contrarily, the use of DFP as iron chelator, caused an increase in TfR1 levels (Fig. 2 D and J) and a decrease of H-ferritin protein levels (Fig. 2 E and K) and of LIP (Fig. 2 F and L). Thus, the RMS cell lines responded to iron modulation as expected.

In summary, the RMS cell lines expressed the main proteins involved in iron trafficking and are able to modulate them controlling the iron uptake (by TfR1) to face the high iron demand of tumor cells for their growth and metabolism and the iron storage (by Ferritins) to protect cells from iron toxicity mediating its storage in a non-toxic form.

### Iron supplementation or deprivation affected RMS cell lines growth

Despite it has been demonstrated that RMS cell lines are able to regulate iron-related proteins after iron modulation (Fig. 2), these tumor cell lines are derived from tissues not canonically involved in iron metabolism/storage, they do not have high basal levels of iron, both as LIP and as in ferritin deposits. Thus, the long exposure to high doses of iron and to an iron chelator could potentially affect the proliferation, growth and migration of these tumor cells.

To this aim RMS cell lines, both EMRS and ARMS were treated with FAC or iron chelators (DFO, DFP, BPS) to test their effect on cell viability.

**Fig. 3:**
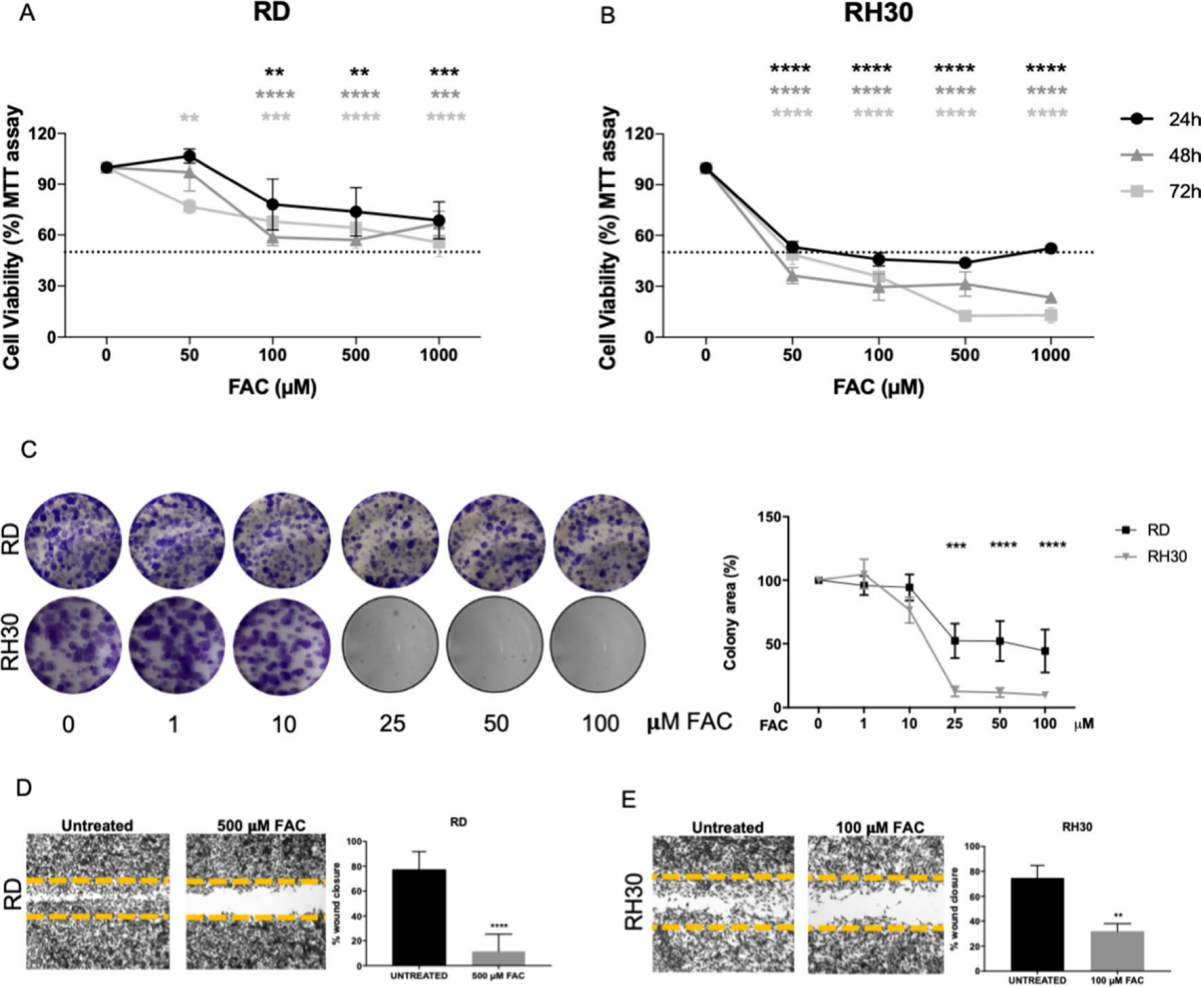
Iron supplementation affected RMS cell viability, clonogenic and wound repair capacity. MTT assay after treatment with 50–100–500–1000 µM of ferric ammonium citrate (FAC) for 24–48–72 h in RD (**A**) and RH30 (**B**) cells, representing human ERMS and ARMS, respectively (*N* = 3). **C** Clonogenic assay in RD and RH30 after treatment with 1–10–25–50–100 µM FAC for 48 h. followed by 5 days without FAC. The representative images showed the formed colonies stained with crystal violet and the correspondent graphs represent the colorimetric quantification of the solubilized crystal violet (N = 3). Wound healing assay in RD (**D**) and RH30 (**E**) after treatment with or without 500 µM FAC for 16 h for RD and 100 µM FAC for 10 h for RH30 (*N* = 3). Statistic was obtained by two-way anova in A and B and Students’ *t* test for unpaired data in C, D, E. The differences were considered as significant for: ****P < 0.0001, ***P < 0.001, **P < 0.01

The treatment with FAC (50–100–500–1000 µM) for 24–48–72 h affected the cell viability of both ERMS and ARMS cell lines already after 24 h exposure, with no substantial differences among the three time points (Fig. 3A, B and Suppl. Figure 1A-B-C-D). The most sensitive RMS cells were RD and RH30, thus further analyses were performed on these two cell lines.

RD cells showed a decrease of about 40% of cell viability with the doses of 100–500–1000 µM of FAC at 24–48–72 h, whereas FAC reduced the RH30 cell viability by 50% already with the dose of 50 µM at 24–48 h and of 70% at 72 h. Higher doses (500–1000 µM) caused even greater reduction of cell viability at 48–72 h (about 70–80% at 48 h and 90% at 72 h), rendering the RH30 cells the most sensitive to iron accumulation (Fig. 3A, B). The IC_50_ was of about 1000 µM at 72 h for RD cells and of about 50 µM at 24–48–72 h for RH30.

We also analyzed the colony formation capacity of RD and RH30 cells after the treatment with lower concentration of FAC (1–10–25–50–100 µM). Iron supplementation caused a dose dependent reduction of colony formation with a 50% reduction for RD cells and 90% reduction for RH30 cells at the doses of 25–50–100 µM, confirming that RH30 were the most sensitive to iron (Fig. 3C).

To determine the effect of iron on cell migration capacity, we performed a scratch on RD and RH30 monolayers. Interestingly, 500 µM of FAC for RD and 100 µM of FAC for RH30 inhibited both cell lines migration by about 80% and 60% for RD (Fig. 3D) and RH30 (Fig. 3E) respectively compared to untreated cells.

On the other hand, iron deprivation could also interfere with the cell proliferation, since iron is an essential trace element for tumor growth. The 24 h-treatment with the iron chelators DFO, DFP and BPS caused a reduction of cell viability of both ERMS and ARMS (Suppl. Figure 1E), with RD and RH30 again the most sensitive ones. Since one of the main oral iron chelators used also in vivo is DFP, we focused the study on this compound.

**Fig. 4:**
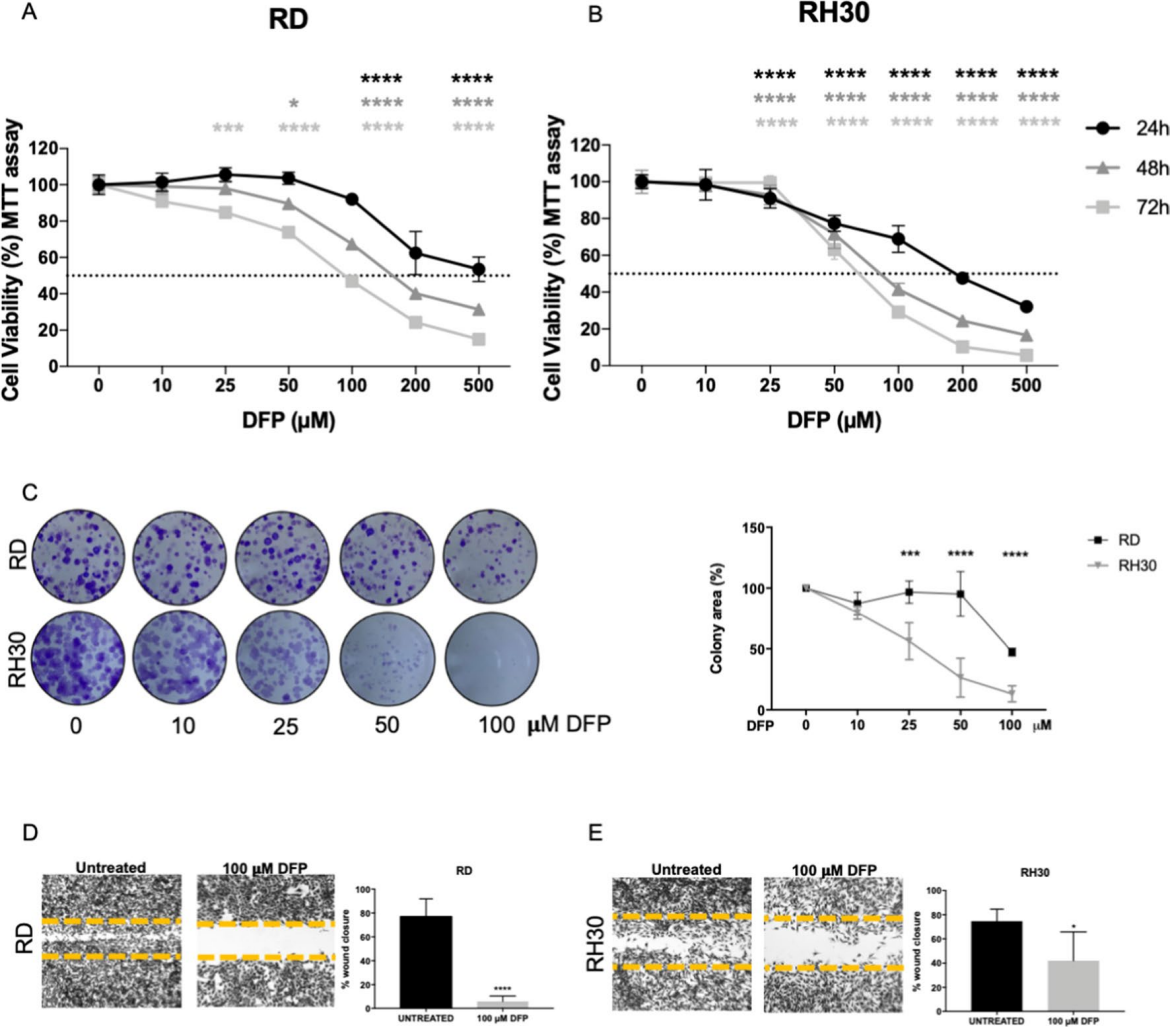
Iron chelation affected RMS cell viability, clonogenic and wound repair capacity. MTT assay after treatment with 10–25–50–100–200–500 µM of Deferiprone (DFP) for 24–48–72 h in RD (**A**) and RH30 (**B**) cells, representing human ERMS and ARMS, respectively (*N* = 3). **C** Clonogenic assay in RD and RH30 after treatment with 10–25–50–100 µM DFP for 48 h followed by 5 days without DFP. The representative images showed the formed colonies stained with crystal violet and the correspondent graphs represent the colorimetric quantification of the solubilized crystal violet (N = 3). Wound healing assay in RD (**D**) and RH30 (**E**) after treatment with or without 100 µM DFP for 16 h for RD and for 10 h for RH30. (*N* = 3). Statistic was obtained by two-way anova in A and B and Students’ *t* test for unpaired data in C, D, E. The differences were considered as significant for: ****P < 0.0001, ***P < 0.001, **P < 0.01

As shown in Fig. 4A, B, RD and RH30 cells were sensitive to DFP in a dose and time dependent manner, with IC_50_ of about 500–150–100 µM at 24–48–72 h respectively for RD cells (Fig. 4A) and an IC_50_ of about 200–90–75 µM at 24–48–72 h respectively for RH30 (Fig. 4B), rendering the last ones the most sensitive to iron chelation.

In addition, RD and RH30 showed a reduced capacity to form colonies in presence of DFP, with a dose dependent decrease. RH30 were again the most sensitive ones, with already a 50% less colonies formed in presence of 25 µM of DFP, whereas RD showed a 50% decrease with the dose of 100 µM (Fig. 4C).

Also, the treatment with DFP was able to prevent RD and RH30 cell migration, with a decrease of 90% (Fig. 4D) and 50% (Fig. 4E), respectively.

### Iron manipulation caused ROS formation and cell death

**Fig. 5:**
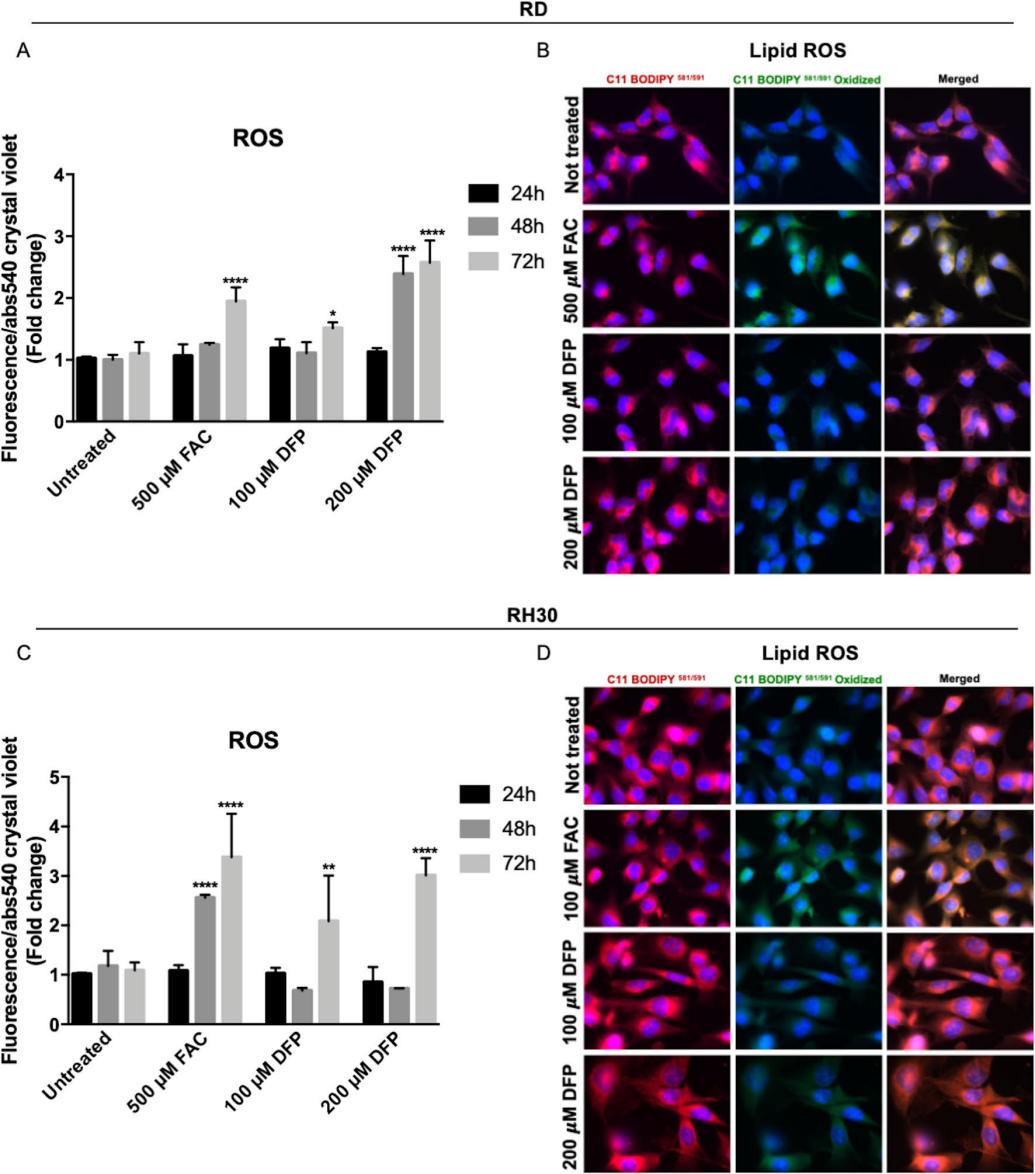
Iron supplementation and chelation-induced ROS formation, but only iron-induced Lipid peroxides formation in RMS cells. RD and RH30 cells treated with 500 µM ferric ammonium citrate (FAC) or 100–200 µM Deferiprone (DFP) for 24–28–72 h. ROS formation in RD (**A**) and RH30 (**C**) was monitored by CM-H2DCFDA probe. The values are expressed as ratio between the fluorescence detected and the absorbance at 540 nm after Crystal violet normalization. RD and RH30 cells treated with 500–100 µM ferric ammonium citrate (FAC) respectively or 100–200 µM Deferiprone (DFP) for 24 h. Lipid peroxides in RD (**B**) and RH30 (**D**) was monitored using C11-BODIPY581/591 probe by a fluorescent microscope. Nuclei were stained with DAPI dye. Images were taken with 63X magnification (with immersion) using different filters (*N* = 3). The differences were considered as significant for: ****P < 0.0001, **P < 0.01, *P < 0.05

To understand the mechanism by which iron modulation, both iron supplementation and its deprivation, caused an alteration of cell proliferation, further analysis has been performed. Since it has been already reported that both iron supplementation and chelation caused ROS formation in other cell lines, the levels of total ROS and Lipid peroxides after FAC or DFP treatment have been analyzed. As reported in Fig. 5A and C both treatments caused the increase in total ROS, with RH30 cells the most sensitive one, as expected. In detail, RD cells showed an increase in ROS (2-fold) after 72 h of FAC exposure (Fig. 5A), whereas in RH30 cells it has been observed a 2.5-fold increase in ROS already after 48 h of FAC treatment and 3.5-fold increase after 72 h of treatment (Fig. 5C). The DFP treatment (200 µM) caused the ROS formation after 48–72 h of exposure in RD cells, with a 2.5-fold increase (Fig. 5A), whereas in RH30 the ROS formation was increased of 2-fold and 3-fold after 72 h of treatment with 100 µM and 200 µM respectively of DFP (Fig. 5C). In addition, it has been observed also an increase of 2 and 4-folds of positive cells to mitochondrial ROS at 48–72 h in RD cells after DFP treatment (Fig. Suppl. 2B).

Interestingly, in both RD and RH30 cells only the treatment with FAC lead to the formation of Lipid ROS, since it has been detected the presence of the oxidized form of the probe (visualized as green fluorescence) (Fig. 5B and D), suggesting a different mechanism of FAC compared to that of DFP.

Since the ROS formation could lead to apoptotic cell death, the Caspases activation has been determined using a commercial multiplexing assay kit for the detection of Caspase-3 (the executioner caspase), Caspase-8 and -9 (the initiators caspases for the extrinsic and intrinsic pathways, respectively) in both RD and RH30 cell lines, after 3–6–24–48–72 h of FAC or DFP treatment.

**Fig. 6:**
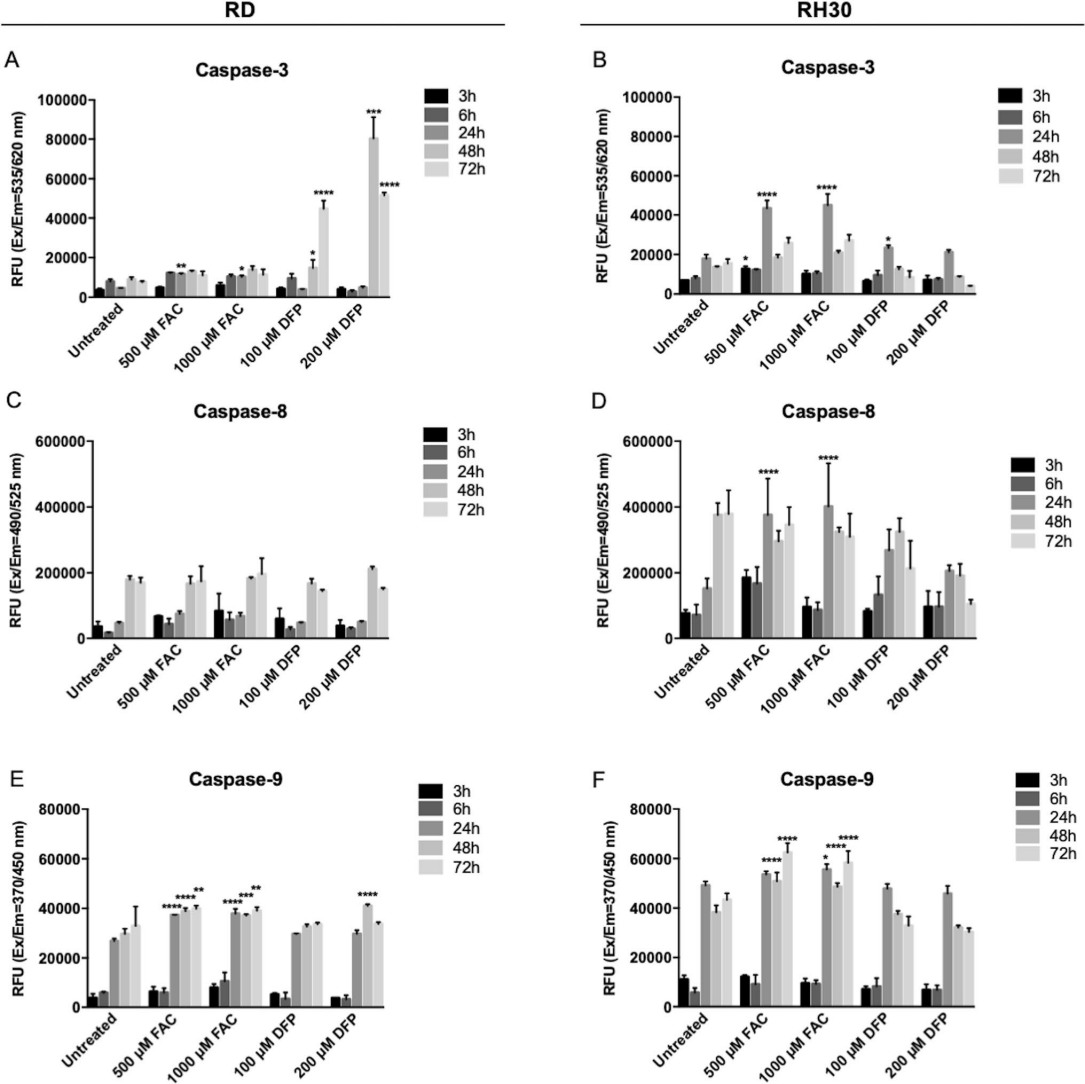
Iron supplementation and chelation-induced Caspase activation in RMS cells. RD and RH30 cells

As shown in Fig. 6, the treatment with FAC culminated in the activation of Caspase-3 in RD and RH30 cell lines (Fig. 6A, B), confirming that the treatments induced apoptotic cell death. In addition, the iron supplementation induced the Caspase-9 activation (Fig. 6E, F), the caspase involved in the intrinsic pathway of apoptosis, which it is usually activated followed by the ROS production, mitochondria damage and the apoptosome formation.

Also, the treatment with DFP induced the Caspase-3 in RD cells (Fig. 6A), statistically significant after 48–72 h of treatment, with the activation of Caspase-9 mainly after 48 h of treatment (Fig. 6E). In RH30 cells, the treatment with DFP caused the activation of Caspase-3 after 24 h-treatment (Fig. 6B) but we did not observe any Caspase-9 activation (Fig. 6F).

On the other hand, the Caspase-8, the initiator of the extrinsic pathway was not affected by both iron supplementation and chelation treatments, in RD (Fig. 6C). Only in RH30 the treatment with FAC after 24 h caused the activation also of Caspase-8 (Fig. 6D).

Finally, the Annexin V/PI assay showed that both the iron supplementation (with 500 µM FAC for 24–48–72 h) and the iron chelation (with 100 µM of DFP for 24–48–72 h)-induced apoptotic cell death in RD cells, with a higher positivity of apoptotic cells mainly after DFP treatment (Fig. Suppl2A).

Treated with 500–1000 µM ferric ammonium citrate (FAC) or 100–200 µM Deferiprone (DFP) for 3–6–24–48–72 h. Caspase-3 (**A, B**), Caspase-8 (**C, D**), Caspase-9 (**E, F**) activation was monitored using a commercial kit (Caspase-3, -8, -9 Multiplex activity assay kit) (*N* = 3). The differences were considered as significant for: ****P < 0.0001, **P < 0.001, **P < 0.01, *P < 0.05.

In summary, iron supplement can cause ROS and Lipid peroxides formation with deleterious effect on cells, causing the inhibition of cell proliferation and the apoptotic cell death. Possibly, due to the Lipid peroxides formation, a component of ferroptotic cell death could also be involved. The DFP cause the formation of ROS and of mitochondrial ROS, leading to profound cell damage and to the apoptotic cell death.

### Iron supplementation and deprivation affected tumor growth in a model of xenograft in vivo

**Fig. 7:**
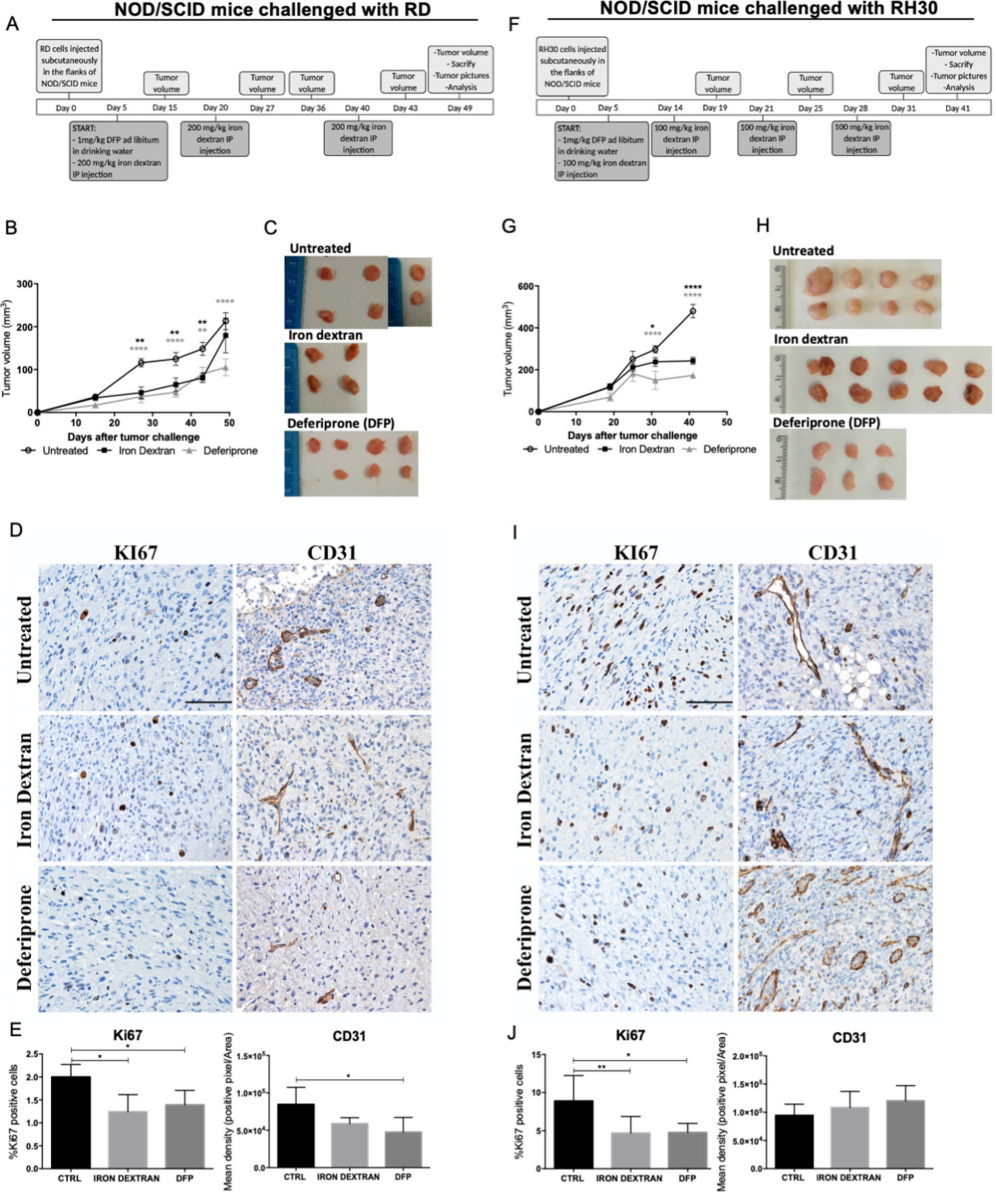
Iron dextran and deferiprone affected the xenograft tumor growth of embryonal RD and alveolar RH30 cells. NOD/SCID mice were inoculated in the flank with 5X10^6^ RD (representing the embryonal RMS) or RH30 (representing the alveolar RMS) cells. 5 days after tumor challenge, mice were randomly divided in 3 groups and treated as reported in (**A**) and (**F**). Tumor dimensions were measured at the time points reported in (**A**) and (**F**). At day 49 (for RD-derived tumors) or 41 (for RH30-derived tumors), mice were sacrificed. Volume (**B** and **G**) and pictures (**C** and **H**) of explanted tumors were recorded. Staining (brown) of KI67 or CD31 was evaluated by IHC analysis on tumor sections. Scale bars: 100 μm. (**D** and** I**). Quantification of the staining was performed considering the % of KI67 positive cells or the Mean density (positive pixel/Area) for CD31. Statistic was obtained by two-way ANOVA for **B** and **G** and by one-way ANOVA for **E** and **J** and the differences were considered as significant for: ****P < 0.0001, **P < 0.01, *P < 0.05

To verify if the treatment with iron supplementation or iron chelation could be a promising approach for RMS, an in vivo experiment was performed. RD or RH30 cells were subcutaneously injected in the flank of NOD/SCID mice and 5 days after the injections, mice were randomly divided in 3 groups and treated with iron dextran (in the form of intraperitoneal injections) or DFP (administrated in drinking water), following the scheme in Fig. 7A and F,

As shown in Fig. 7B and G, the treatments with iron dextran and DFP were effective in inhibiting the tumor mass growth, both derived from RD and RH30, since the tumor volume after treatments was lower than of untreated mice, with the iron chelation the most effective one. The pictures of explanted tumors showed that also at macroscopical level the tumor dimension was significantly reduced after treatment with iron dextran and above all with DFP (Fig. 7C and H).

In addition, immunohistochemical analysis demonstrated that both iron and DFP treatment decreased the number of KI67 positive cells (a marker of cell proliferation) in a statistically significant manner for both RD- and RH30-derived tumors (Fig. 7D–J). Only in RD-derived tumors, the CD31 marker (a marker for angiogenesis) resulted to be significantly decreased after DFP whereas we observed only a tendency after iron challenge (Fig. 7D, E). Contrarily, no differences for CD31 were detected after treatments in RH30-derived tumors (Fig. 7I, J).

The effectiveness of the treatments was confirmed analyzing the iron content in the main tissues involved in iron metabolism, such as serum (Fig. Suppl. 3A and E), liver (Fig. Suppl. 3B and F) and spleen (Fig. Suppl. 3C and G). In addition, the Perl’s staining on spleen, liver and tumors sections confirmed the increase in iron deposits after iron dextran and the decrease after DFP, in all three tissues (Fig. Suppl. 3D and H). The involvement of ferroptosis in the mechanism of tumor growth inhibition by iron found in the in vitro experiments, is an interesting point to prove also in vivo. For this reason, the content of Malondialdehyde (MDA), produced as an end product of lipid peroxidation and considered one of the markers of ferroptosis has been evaluated in tumor xenograft derived from RD or RH30. The analysis showed that only tumors derived from mice treated with iron dextran, has 1.5–2.5-fold increase in MDA, compared to control mice, whereas the DFP treatment did not affect the MDA production (Fig. Suppl. 3I and J), supporting the involvement of ferroptosis.

In summary, considering the effect of both iron overload and iron deprivation in vivo, the iron modulation could be a promising and valid approach to overcome the RMS tumor growth.

#### Discussion

The “iron addiction” is a well-known phenomenon for many cancers, since they require high levels of this metal to sustain their active proliferation [18]. In fact, cancer cells are characterized by elevated iron content due to a deregulation of iron-related proteins such Transferrin Receptor1 (TfR1), Hepcidin, Ferroportin (FPN) and Ferritins (FtH and FtL, essential for iron storage) [18]. However, the high iron content could be also harmful since it leads to a high oxidative stress status, at the limit of the survival. Iron metabolism has been studied in many cancer types, as well as the potential use of iron or iron chelators as therapeutic tools in association to current therapies but not much in Rhabdomyosarcoma (RMS), one of the most aggressive soft tissue sarcomas, which occurs in adolescence and childhood [2].

In the present study, some cell lines representing embryonal (ERMS) and alveolar (ARMS) RMS have been characterized for iron-related proteins and for their sensitivity to iron deprivation or iron supplementation.

Interestingly, we found that both cell lines representing ERMS and ARMS expressed detectable levels of all the iron-related proteins analyzed, such as TfR1, FPN and ferritins, meaning that iron is also essential for their survival and these cell lines are capable to face the proper balance between the iron demand and the iron toxicity, modulating the expression of these proteins.

Since it has already been demonstrated that the excess of iron or its deprivation could be deleterious for many tumor cells due to toxic effect on one hand and the high demand for proliferation and metabolic activities in the other, we also verified the effect of the treatment with increasing dose of iron or iron chelators on RMS cell lines. Interestingly, we found that RD cell line (representing ERMS) and RH30 cell line (representing ARMS) were sensitive to iron supplementation, with RH30 the most sensitive ones, in terms of reduction of cell viability, colony formation and ability to close a wound. The iron excess induced the RMS cell death, likely due to the formation of highly toxic ROS, mitochondrial ROS and lipid peroxides leading to deleterious effect on the cells and with the increase in MDA levels in the tumor xenograft. This is in line with our and other previous works, where it has been demonstrated that RMS cells are significantly sensitive to ferroptosis, a recently discovered form of programmed cell death led by iron, ROS, lipid peroxides accumulation and a reduced efficacy of the antioxidant machinery [27, 28].

The mechanism of action of iron in inhibiting RMS tumor growth seems to involve simultaneously the ferroptotic process (as suggested by the increase in lipid peroxides and MDA) and the intrinsic apoptotic pathway (with the activation of caspase-9 and consequently caspase-3) for both cell lines, with RH30 the most sensitive ones. This could be due to the fact that RH30 are able to respond to iron supplementation, even if in a less efficient manner compared to RD; in fact, upon iron treatment (100 µM FAC), the decrease of TfR1and the increase in H-ferritin was lower than in RD cells whereas the LIP was higher (Fig. 2).

On the other hand, our results clearly showed that RMS cells were more sensitive to iron deprivation with Deferiprone treatment, than to iron supplementation. In fact, DFP caused the formation of highly toxic ROS and consequently the cell death of RD and RH30 cells; moreover, DFP strongly reduced the colony formation and the ability to close a wound in both cell lines. This could be explained by the fact that these cells have a high proliferative ratio thus requiring high amount of iron to sustain their metabolism.

The mechanism of action of DFP seems more complex and, as reported in the literature, cell line dependent. The iron chelators could arrest the cell cycle, induce ROS formation and ER stress leading to apoptosis, affect mitochondria and dysregulate cellular energetics and reverse many hallmarks of cancer cells [29] The treatment with DFP for 48-72 h in RD cells caused an increase in ROS and mitochondrial ROS leading to the intrinsic apoptotic cell death (with caspase-9 and consequently caspase-3 activation and the positivity to Annexin). In RH30 cells, the 72 h treatment with DFP increased the ROS level but we did not observe the caspase activation, even if RH30 cells showed a higher sensitivity to DFP than RD cells, with a lower cell viability (comparing timing and doses between RD and RH30). Likely, the effect of DFP in RH30 cells could be first to the cell cycle arrest accompanied in a second time by the ROS production and cell death, probably caspase independent.

The data obtained in vitro have been confirmed also in vivo, since in the xenograft mouse models with RD and RH30 cell lines implanted subcutaneously, the treatment with iron or the iron chelator Deferiprone significantly reduced the tumor volume compared to the controls, with DFP as the most potent drug. Moreover, the marker of proliferation KI67 resulted decreased after both treatments in both xenografts, corroborating the hypothesis that the treatments inhibited the proliferation of tumor cells.

Among RMS, ARMS is the most aggressive and metastatic one, with an unfavorable prognosis, which often develops resistance to the current therapies. Interestingly RH30 cells, representing ARMS histotype, showed to be highly sensitive to iron deprivation and supplementation giving an alternative therapeutic opportunity, with the iron chelation tool the most effective one both in vitro and in vivo. For these reasons it would be of interest to further investigate the mechanism of action of DFP or other iron chelators in RH30 cells, focusing on the effect on the energetic metabolism, mitochondria functionality and cell cycle arrest.

Interestingly, the use of iron chelators has been already demonstrated to be effective in preclinical studies of other tumors types, such as pancreatic, breast, liver, gastric, hepatic and esophageal cancers [15] and recently, DFP has been proposed as a candidate for drug repurposing and for Phase II clinical trials to overcome Cancer Stem cells (CSC) [30]. Of importance, DFP (known as Ferriprox) is an already FDA-approved molecule for the oral treatment of iron-overload disease (for example in patients affected by thalassemia major) with effective results and low adverse side effects, thus making DFP a suitable and safe drug also for the management of tumor growth, such as of RMS.

In conclusion, these data demonstrated that the iron manipulation, mostly the iron deprivation, seems to be a promising therapeutical tool to overcome RMS tumor growth and it could pave the base for an alternative approach in the management of RMS.

### Supplementary Information

Below is the link to the electronic supplementary material.

Supplementary file1 (TIFF 73688 KB)

Supplementary file2 (TIFF 69481 KB)

Supplementary file3 (TIFF 113794 KB)

Supplementary file4 (DOCX 12967 KB)

## Data Availability

The authors declare that the data supporting the findings of this study are available within the article, its supplementary material and from the corresponding author upon request.
