# Supplementary material for: The modulation of iron metabolism affects the Rhabdomyosarcoma tumor growth in vitro and in vivo
Source: Clin Exp Med. 2023 Feb 10;23(6):2487–502. doi: 10.1007/s10238-023-01012-5 (PMC10543952; doi:10.1007/s10238-023-01012-5)
Supplement: Supplementary file 4 — Supplementary file4 (DOCX 12967 KB) [file 10238_2023_1012_MOESM4_ESM.docx]

**Supplementary Figures**


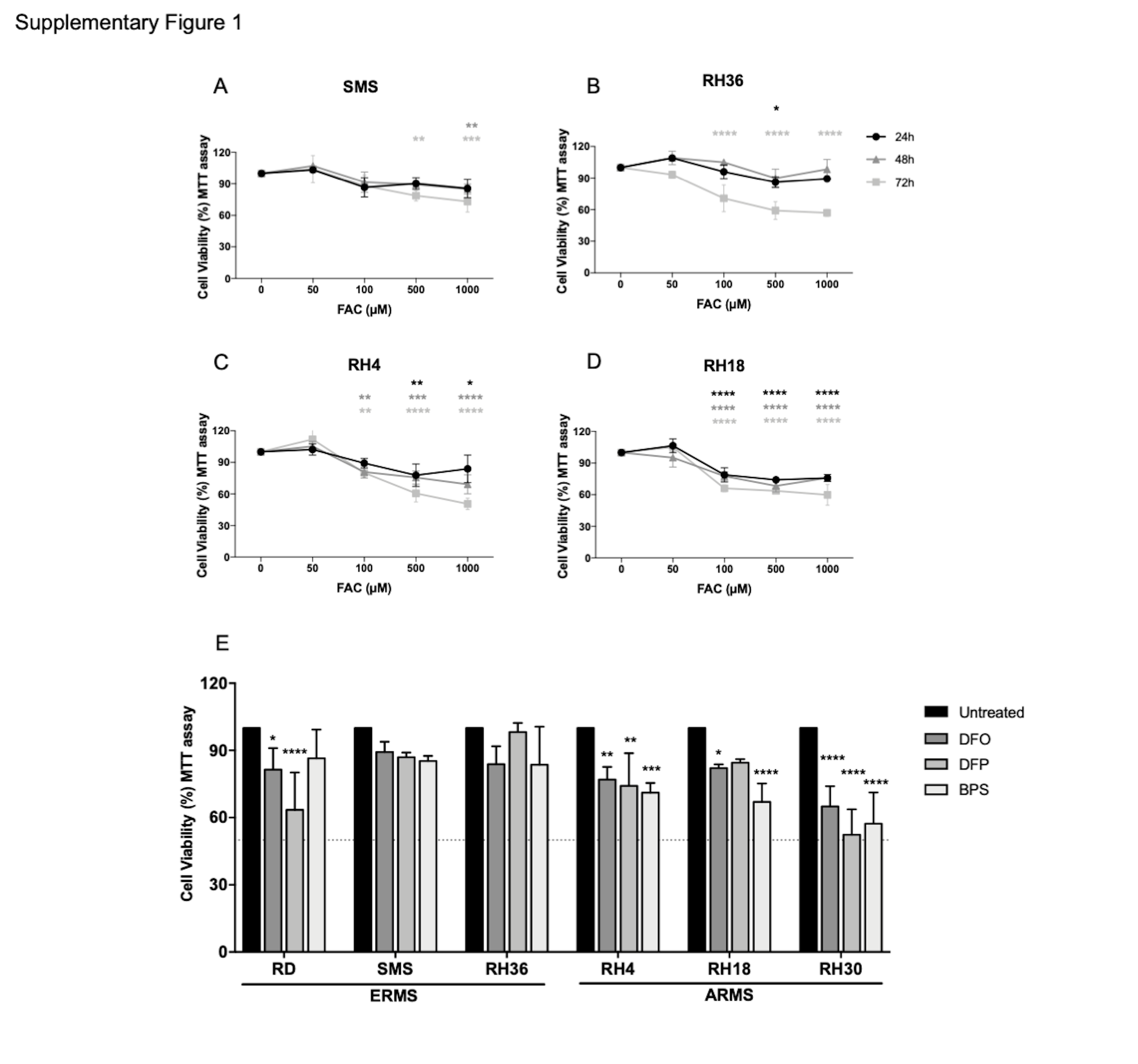


**Supplementary Figure 1. Iron supplementation and deprivation affected cell viability in Rhabdomyosarcoma cell lines**. A-D) MTT assay after treatment with 50-100-500-1000 uM ferric ammonium citrate (FAC) for 24-48-72h in cell lines representing human ERMS (SMS, RH36, A and B) and ARMS (RH4, RH18, C and D). E) MTT assay after treatment with 100 uM the iron chelators Deferoxamine (DFO), Deferiprone (DFP) and Bathophenanthroline disulfonic acid disodium salt hydrate (BPS) for 24h in cell lines representing human ERMS (RD, SMS, RH36) and ARMS (RH4, RH18, RH30). Statistic was obtained by two-way ANOVA. The differences were considered as significant for: *****P <* 0.0001, ****P <* 0.001, ***P <* 0.01, **P <* 0.05.


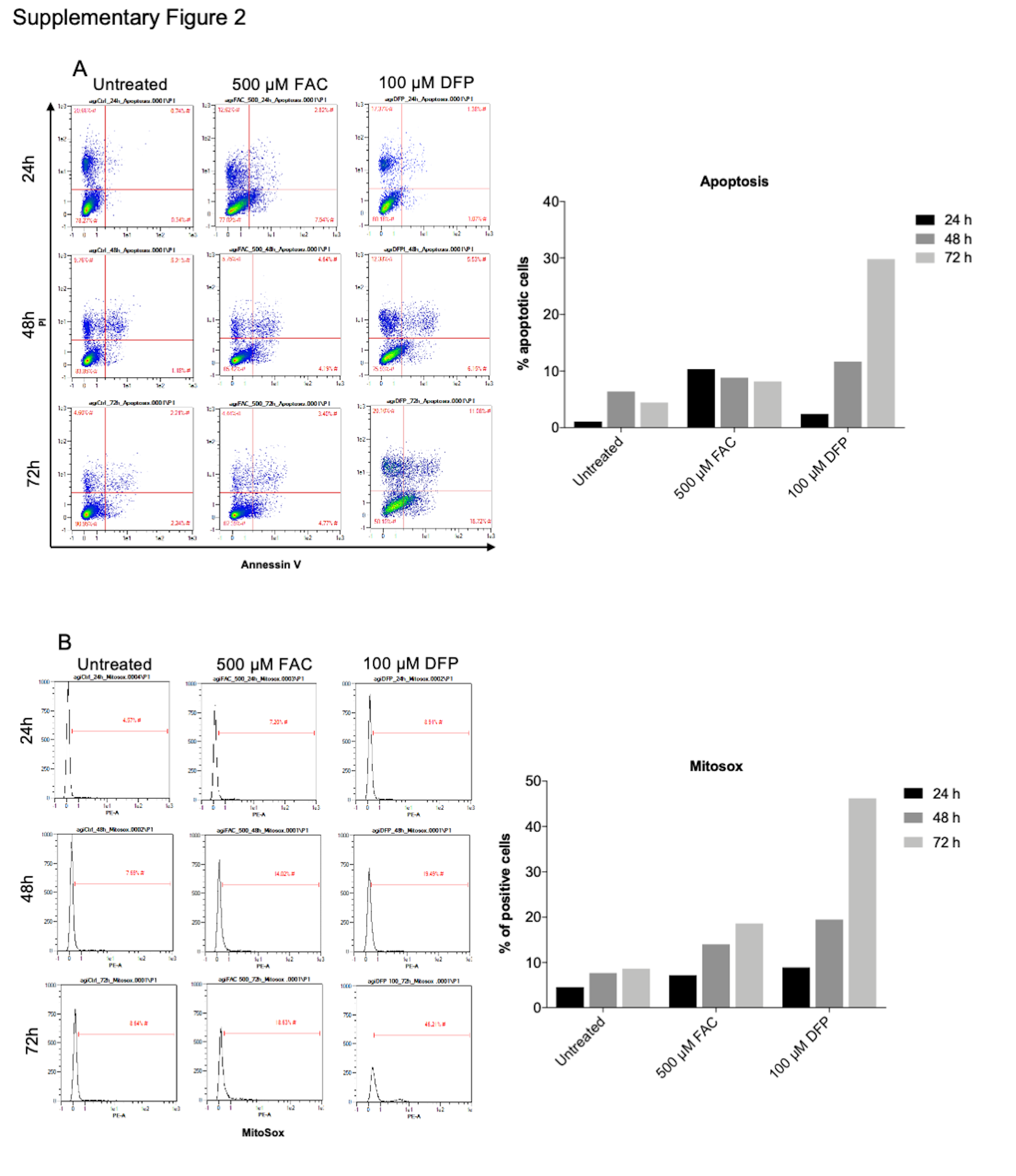


**Supplementary Figure 2. Iron supplementation or deprivation induced apoptosis and mitochondrial ROS formation.** A) RD cells were treated with 500 uM FAC or 100 uM DFP for 24-28-72h and apoptosis was detected by Annexyn V/Propidium Iodide and the positive cells evaluated by FACS. The graph represented the percentage of apoptotic cells. B) RD cells were treated with 500 uM FAC or 100 uM DFP for 24-28-72h and Mitochondrial ROS analyzed by MitoSox assay. The percentage of positive cells, evaluated by FACS, were reported in the graph.


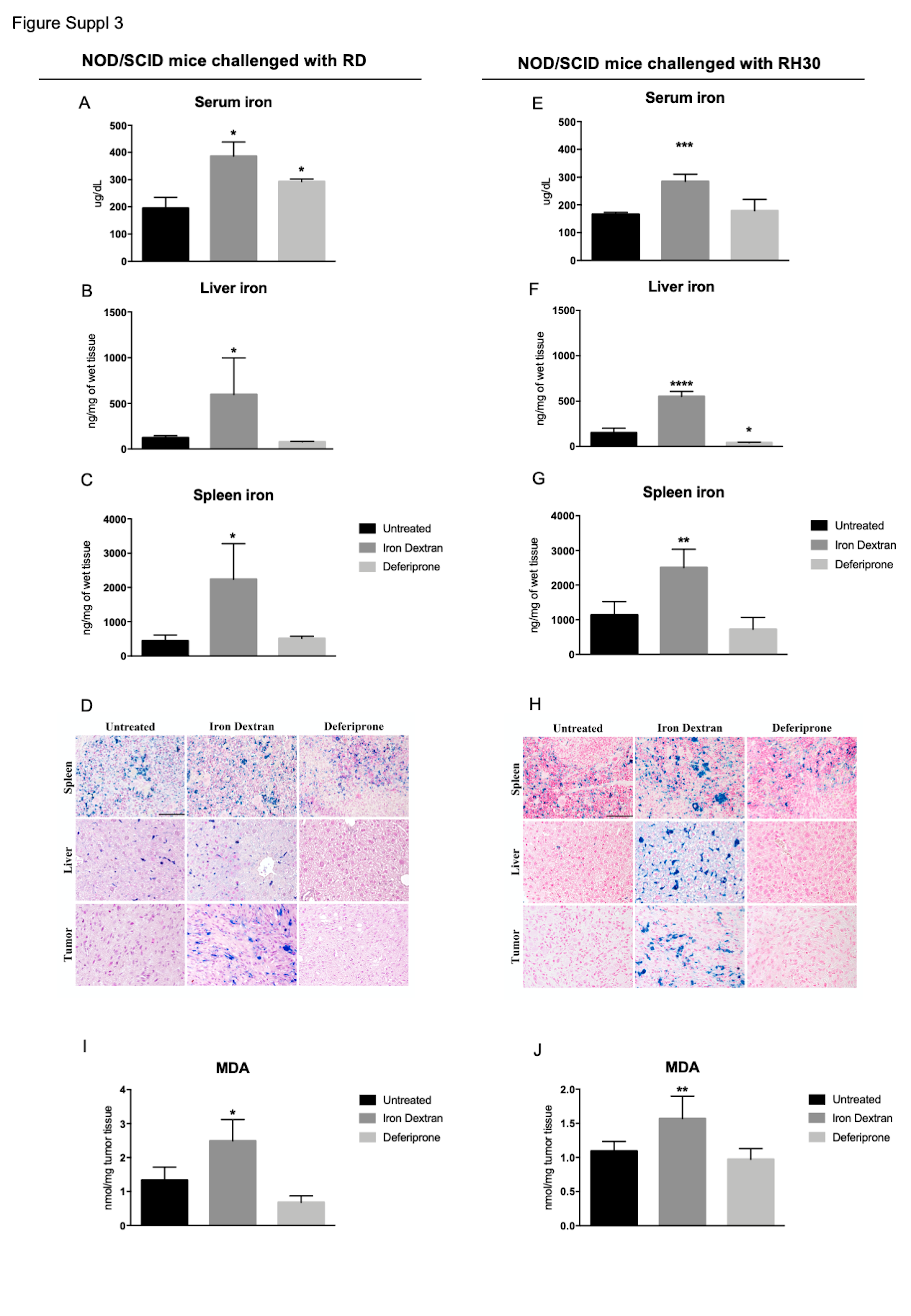


**Supplementary Figure 3. Analysis of iron parameters in tumors and tissues of NOD/SCID mice challenged with the embryonal RD and alveolar RH30 cells.** Iron content in the serum (A and E), liver (B and F) and spleen (C and G) was measured by a spectrophotometric assay. The iron deposits were analyzed by Perl’s staining on tumor, liver and spleen slices and then counterstained by hematoxylin and eosin (D and H). The Malondialdehyde (MDA) content in tumor specimens was evaluated by a commercial kit and expressed as nmol MDA over mg tumor tissue (I and J). Statistic was obtained by one-way ANOVA. The differences were considered as significant for: *****P <* 0.0001, ****P <* 0.001, ***P <* 0.01, **P <* 0.05.
